# Enterovirus infection and its relationship with neurodegenerative diseases

**DOI:** 10.1590/0074-02760220252

**Published:** 2023-03-20

**Authors:** Ivanildo Pedro Sousa, Tuane Cristine Ramos Gonçalves Vieira

**Affiliations:** 1 Fundação Oswaldo Cruz-Fiocruz, Instituto Oswaldo Cruz, Laboratório de Virologia Molecular, Rio de Janeiro, RJ, Brasil; 2 Universidade Federal do Rio de Janeiro, Instituto Nacional de Ciência e Tecnologia em Biologia Estrutural e Bioimagem, Instituto de Bioquímica Médica Leopoldo de Meis, Programa de Biologia Estrutural, Rio de Janeiro, RJ, Brasil

**Keywords:** enterovirus, neurodegenerative diseases, amyotrophic lateral sclerosis, Alzheimer’s disease, Parkinson’s disease

## Abstract

Neurodegenerative diseases (NDs) are increasingly common, especially in populations with higher life expectancies. They are associated mainly with protein metabolism and structure changes, leading to neuronal cell death. Viral infections affect these cellular processes and may be involved in the etiology of several neurological illnesses, particularly NDs. Enteroviruses (EVs) frequently infect the central nervous system (CNS), causing neurological disease. Inflammation, disruption of the host autophagy machinery, and deregulation and accumulation/misfolding of proteins are the main alterations observed after infection by an EV. In this perspective, we discuss the most recent findings on the subject, examining the possible role of EVs in the development of NDs, and shedding light on the putative role played by these viruses in developing NDs.

Neurodegenerative diseases (NDs) are highly debilitating illnesses and the most common causes of severe and fatal dementia. Tauopathies, synucleinopathies, and TDP-43 proteinopathies are the most prevalent NDs and can be categorised primarily by clinical traits and the anatomic distribution of neurodegeneration (Table).^1^ These disorders have pathological hallmarks, including the loss of specific subset neurons in certain brain regions and damage to network synaptic connections that lead to a decline in brain function. Furthermore, recent studies indicate that neuroinflammation is critical for ND pathogenesis and progression.^2^ ^,^ ^3^ Another one of the hallmarks of NDs is the increase in intra- and extracellular amyloid deposits. Amyloid deposits are well-ordered aggregates of misfolded proteins that spontaneously co-assemble into oligomers and reorganise into beta-sheet-rich fibrils.^4^ Additionally, oxidative stress, programmed cell death, and proteotoxic stress associated with changes in the ubiquitin-proteasomal and autophagosomal/lysosomal systems significantly affect the progression of NDs.

Most NDs occur sporadically and are considered multifactorial diseases associated with aging and a complex interaction of epigenetic factors, such as genetics, biological networks (relationships between humans and other organisms), development, and environmental issues.^5^ ^,^ ^6^ Our understanding of how these factors contribute to the development of NDs needs to improve. Numerous studies on NDs and pathogenic organisms have been conducted in recent years, and viruses have mostly been reported as risk factors for the development of neurodegeneration and dementia.^7^ ^,^ ^8^ ^,^ ^9^ ^,^ ^10^ Additionally, emerging evidence suggests that alterations induced in the gut microbiome may play a significant role in the development of NDs. For instance, many viral infections can cause a microbiome-gut-brain axis imbalance (dysbiosis) and are associated with neurodegeneration either directly (through viral infection on neural cells) or indirectly [as the viral infection may trigger a systemic inflammation by increasing gut permeability, which may increase levels of circulating lipopolysaccharides causing the release of inflammatory cytokines in the central nervous system (CNS)].^11^ ^,^ ^12^

**TABLE:** Overview of some neurodegenerative conditions

NDs-types	Main associated disease	Neuropathologies	Protein aggregates
Prion diseases	Creutzfeldt-Jakob disease	Spongiform changes	PrP
Tauopathies	Alzheimer’s disease	Neuritic plaques neurofibrillary tangles	Tau and Aβ
	Pick’s disease	Pick body	Tau
Synucleinopathies	Lewy body disorders	Lewy bodies	α-Synuclein
	Parkinson’s disease	Lewy neurites	
Transactive response DNA-binding protein 43 (TDP-43) proteinopathies	Amyotrophic lateral sclerosis	Motor neuron loss	TDP-43
Inclusion			

## Neurodegenerative mechanisms associated with viral infection

**Fig. 1:**
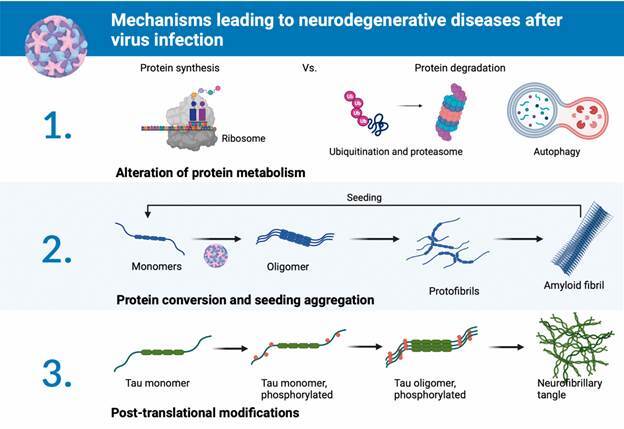
mechanisms leading to neurodegenerative diseases after virus infection. Virus infection can collectively trigger different alterations in cell processes through three main mechanisms: 1. It alters protein metabolism, perturbing protein synthesis and degradation (protein ubiquitination, degradation at the proteasome, and autophagy/aggrephagy). It also affects chaperon prevention/correction of protein misfolding. 2. Viruses can directly interact with proteins, leading to structural conversion, starting aggregation, and forming small toxic aggregates and amyloid fibrils. These fibrils can seed monomer aggregation, representing a self-replicating process. Different virus molecules, such as proteins, lipids, and nucleic acids, are supposed to show this property. 3. Viruses perturb post-translational protein modifications, modulating protein function and oligomerisation. For example, Tau hyperphosphorylation, associated with Alzheimer’s disease, can be triggered by a virus infection, leading to the formation of neurofibrillary tangles and cell dysfunction. This figure was designed at Biorender.com.

The three main mechanisms through which viral infection of the CNS may lead to NDs are protein metabolism alterations, direct conversion and aggregation of cellular proteins by viral factors, and deleterious post-translational modifications (Fig. 1).

Cellular proteostasis results from the action of coordinated networks that maintain a dynamic balance between protein translation, folding, and degradation. Typically, cells avoid forming aggregates, or if they already exist, the cells destroy them in order to prevent any potentially harmful effects.^13^ However, preservation of proteome stability is challenging once cells are exposed to certain environmental stressors. Viral infection is one such stressor whereby the viruses hijack the cellular machinery, alter proteostasis to allow replication and then leave the cell to infect other nearby cells (Fig. 1).

Some viruses can utilise aggregate clearance to increase their replication or evade the immune response.^14^ Heat shock proteins (HSP) such as HSP 40, HSP 70, and HSP 90 have been extensively studied in this area, and they are involved in various stages of viral infection.^15^ HSPs play a vital role in the lifecycle of proteins, including protein folding and refolding, transport, degradation, assembly, activity regulation, and translocation, as well as the depolymerisation of protein aggregates.^15^ The interaction of viral proteins with HSPs can change the apoptosis pathways in which they are involved, making it easier to activate caspase cascades and induce apoptosis to release viruses while evading the immune system.

Additionally, viruses alter the proteasome system to prevent the ubiquitination of viral proteins, leading to their accumulation in the cell.^16^ This scenario, therefore, leads to the accumulation of cellular proteins that also induce their degradation, deposition, and toxicity, rendering cells more vulnerable to protein misfolding. Additionally, antiviral cell responses involving the release of pro-inflammatory cytokines and chemokines can lead to protein misfolding and aggregation, potentially accelerating the onset of genetic forms of NDs and exacerbating preexisting abnormalities.^17^

Viral proteins can interact with various biomolecules inside the cell, directly altering the activity of some proteins and affecting their solubility and stability. In the context of NDs, for example, HIV-Tat (HIV trans-activator of transcription protein) interacts with Aβ peptide *in vitro*, shaping its fibril structure and increasing its toxicity to hippocampal neuron cultures.^18^ Glycoprotein B of the herpes simplex virus (HSV-1) can seed Aβ aggregation, accelerating fibril formation *in vitro*, showing toxicity to neuronal cultures,^19^ and could exacerbate neurodegeneration *in vivo*.

Neurotropic influenza A virus induces the conversion of the cellular prion protein (PrP^C^) into the infectious scrapie prion (PrP^Sc^), and subsequent aggregation, when infecting neuroblastoma cells.^20^ Both nucleic acids (NA) and lipids are also known to induce protein aggregation.^21^ ^,^ ^22^ ^,^ ^23^ ^,^ ^24^ In this context, exogenous NAs are strong candidate inducers that should be considered. Thus, cells may become more prone to protein misfolding due to the interaction of viral factors with host amyloidogenic proteins (Fig. 1). Even if only transient, this interaction may play an important triggering role since these proteins self-perpetuate when aggregated.^25^

Post-translational modification (PTM) is a well-characterised cell strategy to modulate protein function. Nonnative PTM as tau hyperphosphorylation is known to affect its conformation leading to neurofibrillary tangle formation and cell damage (Fig. 1).^26^ HSV-1 infection up-regulates the kinases responsible for Tau phosphorylation (GSK3β-glycogen synthase kinase-3β and PKA-protein kinase A) in human neuroblastoma-infected cells,^27^ leading to hyperphosphorylation and neuronal loss in primary cell culture.^28^ Treatment with acyclovir, which inhibits HSV-1 replication, decreased Tau phosphorylation in cells.^29^ HIV-tat also increases Tau phosphorylation in mice.^30^ Therefore, viral infections can modulate PTMs, thus enhancing neurodegeneration.

It is worth noting that the accumulation and abnormal location of toxic aggregated proteins is one of the clinical hallmarks of neurodegenerative illnesses like Alzheimer’s disease (AD), amyotrophic lateral sclerosis (ALS), and Parkinson’s disease (PD). In this perspective, we present a critical view of the relationship between enterovirus infection and neurodegenerative disorders, shedding light on the putative role played by these viruses in developing NDs.

## Enteroviruses

Globally, enteroviruses (EVs) are one of the major causes of human viral infections. EVs are small, icosahedral-shaped, non-enveloped, positive-sense, single-stranded RNA viruses with a capsid composed of four structural proteins (VP1-VP4) belonging to the genus *Enterovirus* and *Picornaviridae* family. EVs have been classified into fifteen species: EV A-L and human rhinoviruses A-C. However, only EV A to D species and rhinoviruses are known to cause human infection. Due to recurrent mutations and/or recombination, these viruses exhibit a high level of genomic diversity.^31^ While most EVs are transmitted primarily by the faecal-oral route and can advance from the primary site of infection (gastrointestinal tract) to other tissues, some EV-types (e.g., rhinoviruses and EV-D68) can cause respiratory infection and spread via respiratory secretion. Although EV infections in most patients are often mild and self-limiting, these viruses can cause severe CNS illnesses, particularly aseptic meningitis (AM), acute flaccid paralysis (AFP), encephalitis, and acute flaccid myelitis (AFM), which can be fatal. Enteroviruses are the most common cause of aseptic meningitis worldwide. Most of the existing CNS infections associated with EVs have been caused by EV-A71, CVA2, and CVA4 (EV-A species), E6, E11, E30, CVB5 (EV-B species), EV-C99 and CVA24 (EV-C species) and EV-D68 (EV-D species).^32^ ^,^ ^33^

EVs are able to enter into the CNS through a mechanism known as “Trojan Horse” or via neuromuscular junctions using retrograde axonal transport.^32^ ^,^ ^34^ The pathways used by EVs to bypass the blood-brain barrier (BBB) and enter the CNS, the mechanisms of neuronal cell death, and the neuropathogenesis and immunology triggered by EVs during CNS infection have been recently reviewed.^34^ ^,^ ^35^

## The role of enterovirus infection in neurodegenerative diseases

Over the past decades, infections caused by numerous infectious agents have been associated with the pathogenesis of NDs, although this idea is still controversial.^36^ To date, little is known about the potential contribution of enteroviruses in the development of NDs through neuroinflammatory pathways or aggravating the pathogenic alterations that were not necessarily caused by a viral infection, favoring disease progression. EV infection leads to an alteration of the metabolism of the proteins associated with these pathways.

Some studies have highlighted the potential relationship between neurodegeneration induction and AD through Toll-like receptors (TLRs) activation signaling in enterovirus infections. TLRs are a group of receptors that are extensively expressed in several cell types, including immunological, intestinal, lung, and neural cells. They have been linked to inflammatory responses and pathological conditions, including neurodegeneration.^37^ ^,^ ^38^ Additionally, unfolded protein response (UPR) and oxidative stress are two factors that are linked to TLRs and present in different NDs.^38^ ^,^ ^39^ Recently, Luo and coworkers suggested that upon EV-A71 infection, neural cells can trigger the pathogenesis in the brain (mice and human) through the TLR7 pathway inducing neurodegeneration.^37^ Another study found that EV-A71 infection can cause PRSS3 retention in human neuroblastoma SH-SY5Y cells. This essential serine protease acts as a signaling agent and is secreted and transported via the cellular secretory pathway, playing a critical role in the CNS.^40^ PRSS3 accumulation may involve the gain of cytotoxic function. Indeed, enteroviruses infection disrupts the host autophagy machinery at different steps and remodels the secretory pathways inside the cells.^34^ ^,^ ^41^ EV-induced autophagy dysregulation may directly harm cells due to the loss of the protection conferred by autophagy. It is important to highlight the pathogenic roles associated with autophagy impairment in several NDs.^42^

Notably, when infected by EV-A71, neural cells from the mouse brain suffered a depolarisation of aquaporin-4 (AQP4).^43^ In addition to controlling body water balance and water flow in and out of the brain parenchyma, APQ4 may affect several pathways, and decreasing its expression has been closely associated with the development of AD.^44^ The changes brought about by EV-A71 infection on APQ4 may therefore disrupt its ability to clear out beta-amyloid and tau proteins, the accumulation of which is a hallmark of AD and can start years before the disease’s onset.^4^ ^,^ ^43^

Interestingly, recent findings have revealed Aβ to be an antimicrobial peptide, and it has been hypothesised that Aβ deposition may represent an effective innate immune reaction to infection.^45^ Thus, Aβ production may initially be beneficial for defending against microorganisms, but it will become progressively more harmful as the infection becomes chronic or reactivated over time. Regarding the activity of Aβ on EV infection, a recent study has demonstrated that Aβ_1-42_ effectively inhibited EV-A71 in the early stages (attachment and uncoating) of the virus cycle in different cell lines, including neural cells.^46^ Additionally, EV-A71 infection also induced Aβ production and accumulation in SH-SY5Y cells.^46^

**Fig. 2:**
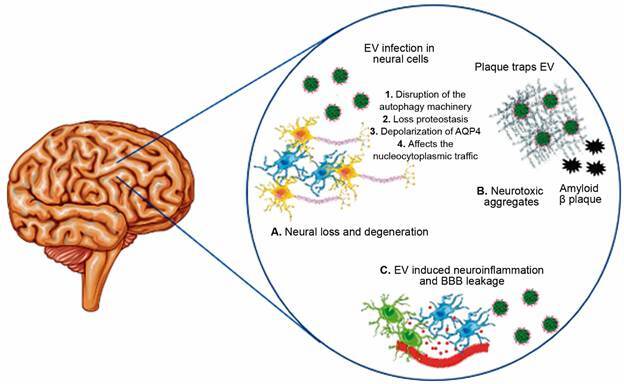
the potential effect of enterovirus (EV) infections in neural cells and its impact on developing neurodegenerative diseases (NDs). (A) After gaining access to the central nervous system (CNS), EV infects neural cells, leading to different alterations in cell processes and decreasing the number of neurons. (B) EV infection could stimulate neural cells to produce amyloid proteins as defense mechanisms, wrapping and neutralising the virus. These amyloid proteins constitute the amyloid plaques, which activate microglia releasing pro-inflammatory cytokines. (C) The blood-brain barrier (BBB) can be disturbed from outside the brain via peripheral inflammatory mediators and dysbiosis, as well as from within the brain by the mediators generated by neural cells that have been stimulated by EV infections.

Several viruses (including enteroviruses) are well-known to be associated with both acute and chronic viral parkinsonism, even though none have been found in Lewy body deposition on examination of *post-mortem* brain tissues; instead, they mimic the signs and symptoms of the disease.^47^ Nevertheless, a few studies have demonstrated that people with a history of poliovirus infection may show an increased risk of developing PD over time.^48^ ^,^ ^49^ Also, in the brainstem neurons of PD patients, virus-like particles and enteroviruses (poliovirus and coxsackievirus) antigens have been identified.^50^ In addition to the direct neuronal cytopathogenic effects of viral infection, these findings suggest that enterovirus infection in PD could directly or indirectly alter the metabolism of α-synuclein. Indeed, a recent study found that α-synuclein is up-regulated and associated with α-synuclein inclusion body formation and dysfunctional autophagy machinery in neurons during coxsackievirus B3 (CVB3) infection.^51^ Additionally, the authors observed α-synuclein aggregates in the cell body of midbrain neurons of mice infected with CVB3.^51^ These modifications might act as triggers for PD. Fig. 2 shows the potential effect of enterovirus infections in neural cells and its impact on developing NDs.

ALS is a neurodegenerative disorder for which the greatest evidence exists linking it to EV infection. Numerous studies have reported EV genome detection in the brain/spinal cord and cerebrospinal fluid of ALS patients, even though the available data are controversial.^52^ ^) and references therein^ Additionally, EV seroprevalence is higher in ALS patients than in controls, regardless of disease progression,^53^ and a high EV-detection rate is observed in spinal cord neurons and CSF of these patients.^54^ ^,^ ^55^ Unfortunately, most of these studies present different EV-detection rates resulting in controversial results, which were not always reproducible,^56^ ^,^ ^57^ likely due to the use of fresh versus archived tissues, the nature of the sample, and methodological differences. However, a recent study found that sublethal infection (mimicking a persistent infection) with CVB3 in ALS genetically susceptible mice resulted in early onset, increased motor impairment, and decreased lifespan.^58^ In addition, pro-inflammatory cytokine/chemokine gene expression increased significantly, independent of immune cell infiltration in the CNS of mice infected with CVB3.^58^ The authors reported that all these modifications induced ALS-related pathologies in the CNS of mice. Interestingly, upon CVB3 infection, TDP-43, a major pathological hallmark in sporadic ALS, is also translocated from the nucleus to the cytoplasm (it is predominantly located in the nucleus), in addition to its decreased solubility and increased cytoplasmic accumulation.^59^ This translocation is observed in patients who have ALS.^60^

## In conclusion

Although EVs are thought to be highly lytic viruses that typically cause acute infections, investigations have shown that EVs can establish a persistent infection in many tissues over time, including neural cells, and can be reactivated either spontaneously or in response to external stimulations.^61^ ^,^ ^62^ Furthermore, chronic EV infection can produce pro-inflammatory cytokines and activate inflammatory reactions in the CNS. These findings imply that a risk factor or cause of NDs may be chronic EV infection.

Overall, although it is widely recognised that EV infections may cause CNS diseases, their involvement in the development of neurodegeneration and dementia, including the mechanisms involved, is not yet fully understood. The recent findings significantly impact most aspects and may suggest an association between the development of NDs and enterovirus infection, although this must be interpreted cautiously. The changes observed during EV infections can lead to a decrease in the number of neurons. The virus-induced damage may exacerbate the effects of typical aging-related neuronal degeneration and thus precipitate the symptoms of ND. Further clinical studies must be conducted to detect EVs in patients with NDs to establish the connection between EV infection and NDs. Causality is difficult to verify since the infection occurs long before clinical signs appear. It will also be interesting to understand the potential impact of EV infection in neural cells, including its role in protein accumulation in secretory vesicles, the disruption of the autophagy machinery, and neuroinflammation, as well as whether persistent EV infection is one of the factors involved in the development of NDs. Finally, EV infection can result in many other health-related disorders, such as diabetes and dysbiosis, which are risk factors for NDs. Therefore, it seems reasonable to investigate the potential relationship between these conditions and viral-induced neurodegeneration. Although significant progress has been made in recent years, more compelling data is still required to link NDs to EV infection.
